# Natural Single-Nucleosome Epi-Polymorphisms in Yeast

**DOI:** 10.1371/journal.pgen.1000913

**Published:** 2010-04-22

**Authors:** Muniyandi Nagarajan, Jean-Baptiste Veyrieras, Maud de Dieuleveult, Hélène Bottin, Steffen Fehrmann, Anne-Laure Abraham, Séverine Croze, Lars M. Steinmetz, Xavier Gidrol, Gaël Yvert

**Affiliations:** 1 Laboratoire de Biologie Moléculaire de la Cellule, Ecole Normale Supérieure de Lyon, CNRS, Université de Lyon, Lyon, France; 2 ProfileXpert, Bron, France; 3 Genome Biology, European Molecular Biology Laboratory, Heidelberg, Germany; 4 Laboratoire d'Exploration Fonctionnelle des Génomes, Commissariat à l'Energie Atomique, Direction des Sciences du Vivant, Institut de Radiobiologie Cellulaire et Moléculaire, Evry, France; University of Michigan, United States of America

## Abstract

*Epigenomes* commonly refer to the sequence of presence/absence of specific epigenetic marks along eukaryotic chromatin. Complete histone-borne epigenomes have now been described at single-nucleosome resolution from various organisms, tissues, developmental stages, or diseases, yet their intra-species natural variation has never been investigated. We describe here that the epigenomic sequence of histone H3 acetylation at Lysine 14 (H3K14ac) differs greatly between two unrelated strains of the yeast *Saccharomyces cerevisiae.* Using single-nucleosome chromatin immunoprecipitation and mapping, we interrogated 58,694 nucleosomes and found that 5,442 of them differed in their level of H3K14 acetylation, at a false discovery rate (FDR) of 0.0001. These Single Nucleosome Epi-Polymorphisms (SNEPs) were enriched at regulatory sites and conserved non-coding DNA sequences. Surprisingly, higher acetylation in one strain did not imply higher expression of the relevant gene. However, SNEPs were enriched in genes of high transcriptional variability and one SNEP was associated with the strength of gene activation upon stimulation. Our observations suggest a high level of inter-individual epigenomic variation in natural populations, with essential questions on the origin of this diversity and its relevance to gene x environment interactions.

## Author Summary

Nucleosomes are the basic units of chromatin, with part of the long DNA molecule wrapped around a multiprotein core, which makes unpacked chromatin often portrayed as a string of pearls. This string can carry three types of sequences: DNA, methyl groups on cytosines, and, on every pearl, the presence-or-absence of histone post-translational modifications such as acetylation of lysines (nucleosomal epigenome). These latter sequences can change dynamically, and the mechanisms involved are heavily studied as they participate in many physiological processes (pluripotency, disease…). However, nothing is known about the natural diversity of nucleosomal epigenomes in natural populations. As a model, we compared two unrelated yeast strains for their epigenome of one histone modification. We found a high divergence, which was enriched at regulatory sites and often carried on specific nucleosomes. Although this nucleosome modification is usually associated with high transcription, higher acetylation in one strain did not necessarily imply higher expression of the corresponding gene. However, one nucleosomal variation was associated with a stronger gene activation upon stimulation. These results suggest that nucleosomal epigenomes largely differ between individuals, raising essential questions on the origin of these differences and their contribution to personal responses to environmental changes (such as clinical treatments).

## Introduction

Divergence of DNA sequences between individuals has been the basis of genetics for half a century. More recently, epimutations were identified where inter-individual differences resided in DNA methylation patterns rather than in the DNA sequence itself, with notable consequences on imprinting and phenotypes [1]–[4]. In addition to DNA methylation, nucleosome positioning and post-translational modifications of histone tails have received increasing interest as they can regulate gene activity and genome dynamics [5]. A wealth of stimulating research has been conducted on these modifications, leading to a more and more precise characterization of the machineries remodeling them (such as acetyl- or methyl-transferases), of the pathways regulating these machineries (such as environmental cues), of the factors recognizing these modifications (such as bromo- and chromo-domain containing proteins), and of the consequences of these interactions on cellular outcomes (such as cellular differentiation or disease) [6]. In addition, the genomic distributions of these histone marks have been described in various organisms and cell types [7]–[11], raising the hope to understand or predict outcomes of eukaryotic cells from the sequence of their *epigenomes*. Many laboratories are therefore intensively studying if and how information can be coded by epigenomes [12], [13].

Whether epigenomic sequences vary in natural populations has only been poorly investigated. Recent studies showed a rather abundant natural epigenetic variation of methylated DNA in plants, which was shown to correlate to transcriptional differences and to be additively inherited [3], [14]. In addition, cases of allele-specific histone modifications have been reported [15]–[17]. But a detailed comparison of histone-tail epigenomes has been lacking.

Using two unrelated strains of the yeast *Saccharomyces cerevisiae* as a model system, we provide here a first estimate of this variability for one histone post-translational modification at a single-nucleosome resolution. The number of epi-polymorphisms was high, with notable enrichment in regions of conserved DNA sequences and numerous cases where a precise (isolated) nucleosome was targeted. This variability was not correlated to differential transcription but to the degree of transcriptional response to perturbations. Our observations provide a basis for population epigenomics and raise essential questions on the origin of this diversity and its contribution to inter-individual variability in the response to environmental changes.

## Results

### Genome-wide nucleosome positioning is largely conserved between two unrelated *S. cerevisiae* strains

**Figure 1:**
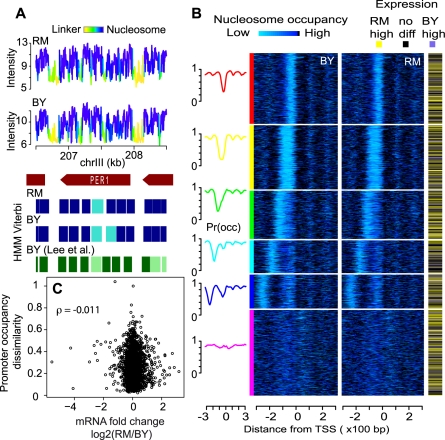
Nucleosome positioning in two unrelated natural *S. cerevisiae* strains. (A) Example of raw signals and nucleosome positioning inference in the region of the *PER1* gene. Nucleosomal DNA was purified from each strains in triplicate, amplified linearly and hybridized to whole genome oligonucleotide Tiling arrays. Data were log-transformed and normalized using the quantile-quantile method and averaged across replicates to produce the probe-level signal intensities shown on the top panels. A Hidden Markov Model (HMM) similar to the one previously described [25] was applied to each strain independently to infer nucleosomal positioning (blue rectangles). Faded and plain colors represent ‘delocalized’ and ‘well-positioned’ nucleosomes, respectively, as defined previously [24]. Signal intensities are colored according to the HMM posterior probability to be within a nucleosome (cumulating delocalized and well-positioned). Nucleosome positions from the published atlas of Lee et al. [24], who used a strain isogenic to BY, are indicated by green rectangles and are also faded when reported as ‘delocalized’. (B) Genes (rows) were clustered based on profiles of nucleosome occupancy at their promoter in the BY strain (see Methods). Their order was then used to plot heatmaps of nucleosome occupancy around transcriptional start site in BY and RM, respectively, as well as expression divergence between the two strains (according to statistical significance at FDR 5% from the dataset of Brem et al. [20]). Left curves represent mean occupancy profiles of the six main classes of promoters. (C) Absence of correlation between promoter occupancy and expression divergence. Each dot represents one gene. X-axis: inter-strain difference in expression measured as log2(RM/BY) from Brem et al. [20]. Y-axis: inter-strain dissimilarity of promoter occupancy profiles. For each promoter region, the RM/BY dissimilarity was estimated as 1 - R, where R is the Spearman correlation coefficient between the BY and RM occupancy profiles shown in (B). ρ: Spearman correlation between the resulting X and Y data.

To provide a first estimate of nucleosome-level epigenomic diversity, we used two unrelated strains of the yeast *S. cerevisiae* (BY and RM) as a model system. These strains were previously used to investigate natural genetic variability within *S. cerevisiae* for various phenotypes such as cellular morphology, sensitivity to drugs, gene expression or telomere length [18]–[21]. BY is a commonly used laboratory strain, it is isogenic to S288c which derives from a clone isolated from a rotten fig in California. RM (also called RM11-1a) derives from an isolate collected in a Californian vineyard by Robert Mortimer [22]. We compared them with respect to nucleosome positions as well as epigenomic sequence of one histone tail modification. Nucleosome positions were mapped using whole-genome 4-bp resolution tiling microarrays [23], [24]. Of the 6,553,600 probes of the microarray, 2,801,885 and 2,570,638 had a single perfect match on BY and RM genome, respectively. Only signals from these probes were used for analysis, averaging ∼34 reliable probes per nucleosome. We aligned the two assembled genome sequences and used probe positions to fit a Hidden Markov Model (HMM) for inference of nucleosome positioning in each strain, as previously described [25] (Figure 1A). Note that the HMM algorithm was run on BY and RM datasets independently. Positioning looked similar between the two strains and was in very good agreement with a previously published atlas of positions [24]. We systematically aligned nucleosomes between the two strains (see Methods) and found that positioning was generally well conserved: The distance between BY and RM midpoints was smaller than 19 nucleotides in 75% of all nucleosomes; and the overlap covered at least 78% of BY nucleosome length in 80% of alignments (Figure S1). Nucleosomal occupancy was also conserved except at specific regions near heterochromatin sites (telomeres and rDNA repeat) (Figure S2). Comparison of SNP densities in linkers versus nucleosomal DNA was consistent with the results obtained when using the atlas of Lee et al. [24] (Text S1).

We examined in more details occupancy around transcription start sites and found the stereotyped nucleosome-depleted regions flanked by well-positioned nucleosomes (Figure 1B and Figure S3). The typical nucleosome depletion at transcription end sites [26] was also observed in both strains (Figure S3). We clustered promoters according to their nucleosome signature in the BY strain only, and used the resulting gene order to plot occupancy data in BY and RM as heatmaps, as well as differential gene expression known from previous studies (Figure 1B). The similarity of the occupancy profiles of the two strains contrasted with the large extent of transcriptional differences (Figure 1B and 1C).

### Inter-strain comparison of an epigenomic sequence reveals abundant nucleosome-level epi-polymorphisms

We then searched for nucleosomes bearing differential levels of a specific histone post-translational modification. By analogy to nucleotide polymorphisms, we called these nucleosomes ‘Single Nucleosome Epi-Polymorphisms’ (SNEP). We chose acetylation of lysine 14 of histone H3 because it was reported to be largely distributed over the genome and not restricted to specific regulatory positions [9], [27]. Hereafter, ‘BYac’ and ‘RMac’ SNEP will refer to nucleosomes where H3K14 is preferentially acetylated in BY and RM, respectively. To detect such nucleosomes, we used ChIP-CHIP [7] and we developed a custom algorithm for data analysis. First, only probes that had a single perfect match on both BY and RM genomes were retained. This precaution is important as DNA polymorphisms can greatly affect hybridization intensities. For each pair of aligned nucleosomes, probes that were not entirely covered by both BY and RM nucleosomes were also removed and a dedicated analysis of variance was applied (see Methods). The underlying linear model integrated both nucleosome mapping and chromatin immuno-precipitation experiments, which enabled to decouple the call for SNEPs from strain differences in occupancy intensity.

**Figure 2:**
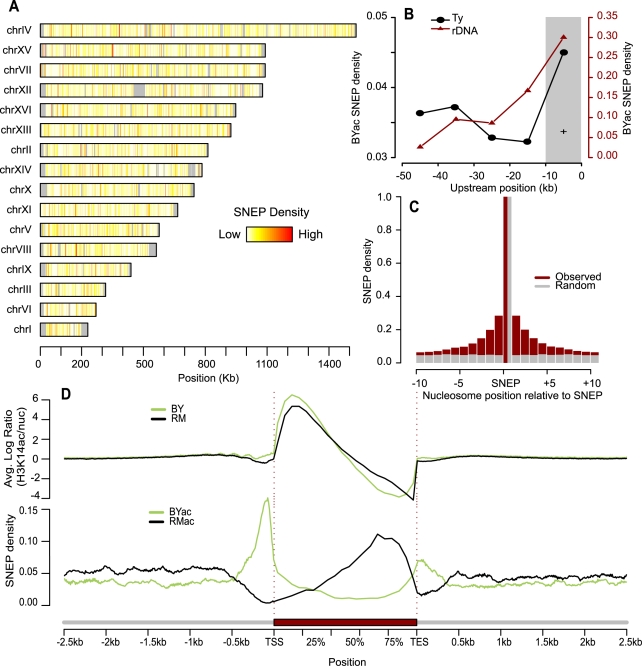
Abundance and genomic distribution of Single Nucleosome Epi-Polymorphisms. (A) The fraction of nucleosomes that were called SNEP at FDR  = 0.0001 was computed in every 1Kb-segment along each chromosome. Density ranged from 0 (white) to 100% (red). Grey denotes regions where nucleosomes could not be aligned. (B) Enrichment of H3K14ac SNEPs upstream Ty insertions and rDNA repeats. The fraction of BYac SNEPs among all nucleosomes was counted in 10 kb intervals upstream the rDNA region (brown triangles). The 7 fold enrichment of BYac SNEPs in the first 10 kb was significant (grey area, Chi-square test *P* = 0.01). Upstream regions of all Ty insertions present in BY and absent from RM were analyzed similarly (black points), and their fractions of BYac SNEPs were averaged. The 1.3 fold enrichment in the 10 kb interval directly upstream the insertions was significant (grey area, Chi-square test *P* = 0.014). (C) Local correlation between H3K14ac SNEPs. Ten nucleosomes were interrogated upstream and downstream each SNEP (x-axis). For each one, cases where the nucleosome was a SNEP similar to the centered one (either BYac or RMac) were counted and divided by the total number of nucleosomes interrogated at that position (brown histogram). Control values were obtained from the same procedure applied after re-assigning SNEPs to random nucleosomes (grey histogram). (D) Density of H3K14 acetylation and SNEPs relative to gene position. Every gene was divided by segmenting the coding sequence in 10 bins (average bin size of 160 bp) and its upstream and downstream regions in 100 bp bins. For every gene and every bin, *log(acBY/nucBY)* was averaged across replicated experiments and across all probes matching intra-nucleosomal DNA to produce the top green profile. Similarly, averaged *log(acRM/nucRM)* values generated the top black profile. Here *acBY* and *acRM* refer to H3K14ac ChIP-CHIP experiments on BY and RM samples, respectively, while *nucBY* and *nucRM* refer to nucleosomal mapping experiments on BY and RM samples, respectively. Note that probes matching inter-nucleosome linkers do not contribute to the profiles, which are therefore corrected for nucleosome abundance. Bottom profiles were obtained by counting the fraction of BYac SNEPs (green) and RMac SNEPs (black) among all nucleosomes that overlapped at least partially the bin, and averaging these fractions across all genes.

This method identified 5,442 H3K14ac SNEPs at nominal *P*-value <9.27×10^−6^ which corresponded to a False Discovery Rate [28] (FDR) of 0.0001. This list was used in all further analysis described here. SNEPs were distributed all over the genome, with few particular hotspots (Figure 2A). Epigenetic variability was very high, as these highly significant SNEPs were found in nearly 10% of nucleosomes interrogated. At the commonly used level of FDR  = 0.01, 25.3% of nucleosomes were significant SNEPs, and further relaxing the detection threshold to FDR  = 0.2 listed 31,854 SNEPs. We can therefore assume that about 40% of the chromatin is variable for this epigenetic mark between the two strains. In most cases, SNEPs were not detected as all-or-none nucleosomal acetylation, but as a quantitative difference between the two strains. The degree of inter-strain difference varied between SNEPs (Figure S4), with most cases displaying a 1.2 to 1.5 fold difference. Intriguingly, the acetylation difference was more pronounced in BYac SNEP (918 SNEPs at >1.5 fold) than in RMac SNEPs (274 SNEPs at >1.5 fold).

### Genomic distribution of SNEPs

Because some highly polymorphic DNA features are associated with chromatin silencing, specific cases of histone acetylation epi-polymorphisms could be expected. One example is the rDNA locus, a repetitive sequence silenced by the Sir2 histone deacetylase [29], which is 15.6 Kb longer in RM than in BY. This higher repeat length could better recruit deacetylase activity and generate BYac SNEP in the vicinity of the repeat. Consistently, we saw a significant enrichment of BYac SNEPs in the region directly upstream rDNA (Figure 2B). Other examples are Ty retrotransposons. They differ greatly between natural strains, their epigenetic effect on nearby gene expression has long been observed [30] and their active LTR promoters are known to recruit the SAGA histone acetyltransferase [31]. Thus, nucleosomes residing near a Ty element in one strain but not in the other may harbor acetylation epi-polymorphisms. Consistently, BYac SNEPs were significantly enriched near BY Ty insertions (Figure 2B).

If such large position effects were the general source of H3K14ac epipolymorphisms, one would expect SNEPs to cluster together at particular hot spots. We clearly observed local correlations, as epipolymorphisms were 7 times more frequent than expected by chance among nucleosomes adjacent to SNEPs, and this effect could span over 10 nucleosomes upstream and downstream of a SNEP (Figure 2C). However, the majority (55%) of SNEPs were limited to a single nucleosome. This is unlikely to be a detection limitation, as 994 SNEPs had both flanking nucleosomes still scoring non-polymorphic at *P*<0.01. Thus, local correlation seems to be limited and epi-polymorphisms are frequently distributed on specific nucleosomes.

SNEPs were not uniformly distributed along genes. The averaged H3K14 acetylation profile of both strains was consistent with previous descriptions [7], [27], with enrichment downstream transcription start sites and decreased acetylation at the end of transcribed sequences (Figure 2D). However, strikingly, BYac SNEPs were abundant upstream TSS and around TES, while RMac SNEPs marked the second half of transcribed regions. These patterns were also visible when selecting only SNEPs with strong effect (>1.4-fold acetylation difference). This could result from a better recruitment of Rpd3S deacetylase behind elongating RNA polymerase II [32], as signs of elongation impairments were previously seen in RM [33]. It is important to note that this pattern of SNEP distribution reflects an average tendency, and that several genes present a totally different epigenetic pattern. For example, the *NDE2* gene did not have BYac SNEP in promoter nor in terminator region, but had RMac SNEPs at the beginning of its coding region (Figure S5). Finally, RMac SNEPs were slightly more frequent than BYac SNEPs (58.5% versus 41.5%).

### SNEPs are not correlated to differential transcription levels

**Figure 3:**
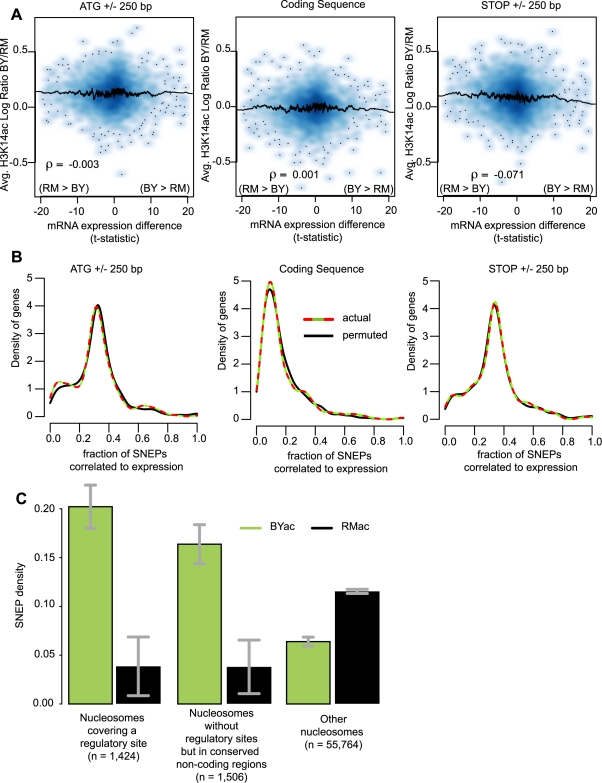
SNEPs are not associated with transcriptional differences but are enriched at conserved regulatory sites. (A) Display from microarray data directly. Density plots representing the distribution of genes with respect to H3K14 acetylation differences (y-axis) and gene expression differences (x-axis). For every gene, three regions were considered as indicated above the panels. For each region, H3K14ac inter-strain difference was estimated as *log(acBY/nucBY)−log(acRM/nucRM)* (as defined in legend of Figure 2D), averaged across replicated experiments and across all probes interrogating nucleosomal DNA of the region. Gene expression inter-strain differences are represented by their *t*-statistic computed from data of Brem et al. [20]. ρ, Pearson correlation coefficient. A similar picture was obtained when using fold change of expression instead of *t*-statistics (Figure S10). (B) Display from SNEP locations. For every gene, the fraction of H3K14ac SNEPs correlated to expression was defined as the number of SNEPs acetylated in the strain with highest expression, divided by the total number of nucleosomes in the region. Curves represent the density distribution of genes according to this measure, from actual data (colored) and data where indexes of expression ratios were permuted (black). Colored curves are not significantly shifted to the right (as compared to black curves), ruling out association between SNEP and gene expression differences. (C) BYac but not RMac SNEPs are more abundant at conserved regulatory sites. Nucleosomes were divided in three categories: nucleosomes that covered entirely a conserved regulatory site from the list of MacIsaac et al. [35], nucleosomes that did not contain such sites but were located in highly conserved non-coding sequences (see Methods), and nucleosomes excluded from the first two categories. The fraction of SNEPs within each category is presented. Error bars, 95% C.I. The 3.2 and 2.6 fold enrichment at regulatory sites and other conserved regions, respectively, were highly significant (*P*<2.2×10^−16^).

Since acetylation of H3K14 is known to be associated with high transcription levels [7], [27], [34], its inter-strain variability could simply reflect inter-strain differences in gene expression. Transcriptional variation between BY and RM has been extensively studied in the same growth conditions as here [20], thus allowing direct examination of this possibility. We considered three regions at the beginning, middle and end of genes, and computed in each one the average log-ratio of H3K14 acetylation between the two strains. In all three regions this ratio was clearly not correlated to expression differences (Figure 3A). Consistently, SNEPs acetylated in the strain with highest gene expression were not over-represented in any of the three regions (Figure 3B). The two strains therefore have a high degree of divergence at both transcriptomic and epigenomic levels but with no apparent connection between the two.

### BYac SNEPs are more abundant in conserved regulatory regions

If not correlated to expression differences, do SNEPs have any functional implication? If so, one might expect them to target nucleosomes located at critical positions for gene regulation, such as nucleosomes containing a transcription factor binding site. In favor of this, and in accordance with the distribution pattern described above, we found a striking (3.2-fold) enrichment of BYac SNEPs in nucleosomes that fully covered a conserved regulatory site [35] (Figure 3C). BYac SNEPs were also abundant in conserved non-coding regions regardless of regulatory sites (Figure 3C). In contrast, RMac SNEPs were poorly present at these conserved regions (Figure 3C), which is consistent with their enrichment within protein coding regions (Figure 2D). The abundance of BYac SNEPs at conserved regulatory sites indicates that genetic and epigenetic polymorphisms can be complementary, the latter providing diversity where the former is more constrained.

### SNEPs are enriched in genes with high expression variability

Are genes with high inter-strain variability in gene expression the same genes as those having high epigenomic variability? Although SNEP acetylation was not associated to higher gene expression, it remained possible that genes with high expression changes contained more SNEPs than others. We examined this possibility by ranking genes according to their BY/RM expression fold-change and by counting their SNEP content (Figure S6A). This showed that indeed, SNEPs were more frequent in genes showing high inter-strain transcriptional differences.

Several studies have examined the evolvability of yeast gene expression levels. For example, when comparing 4 yeast species across 5 different stressful environments, Tirosh et al. showed that genes can have very different inter-species expression divergence [36]. Similarly, Landry et al. showed that *S. cerevisiae* genes greatly differ in their divergence of expression across independent mutation accumulation lineages [37]. To see if SNEP abundance was correlated with expression evolvability beyond the scope of the BY and RM strains, we used these datasets to rank genes either by their expression divergence [36] or by their mutational variance [37]. This showed an unambiguous association between SNEP frequency and expression variability (Figure S6B and S6C).

The extent of gene x environment interactions in the control of gene expression has been thoroughly estimated by Smith et al. who used the same BY and RM strains as here and compared their transcriptomes between two different steady-state environments: growth in glucose and growth in ethanol [38]. Using this dataset, we examined if SNEP frequency in genes was associated with the level of genotype x environment interaction in the gene's expression level (Figure S7). We found that BYac but not RMac SNEPs were more frequent in genes with high genotype x environment interaction, with no correlation between the direction of the SNEP (which strain is acetylated) and the direction of the interaction (which strain shows the highest change between glucose and ethanol growth). SNEP acetylation was therefore not predictive of the amplitude of expression change between the two different environments. However, it is important to note that these two environments were stable and this dataset did not correspond to the dynamic response to an environmental change.

**Figure 4:**
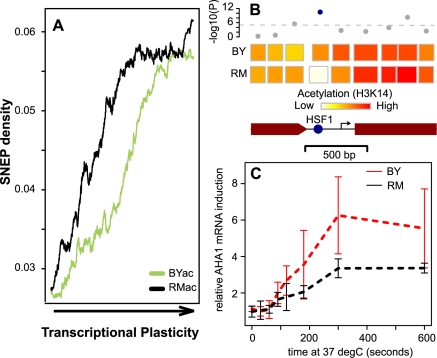
SNEP correlation with transcriptional plasticity. (A) SNEP density is correlated to transcriptional plasticity genome-wide. 4,232 genes were ranked according to their plasticity values from Tirosh et al. [36] on the x-axis. BYac and RMac SNEP frequencies were counted among nucleosomes located in each gene (coding region plus and minus 250 bp), and averaged in 500-genes sliding windows (y-axis). (B) Scheme of nucleosome organization in the regulatory region of the *AHA1* gene. Rectangles represent nucleosomes, colored according to the mean *log(ac/nuc)* value across all probes of the nucleosome. SNEP detection (-log_10_(P-value)) is indicated above each nucleosome, with significance cutoff indicated as a dashed line. A highly significant SNEP covers the DNA binding site for HSF1 transcription factor (blue spot). Arrow, transcription start site. Brown box, beginning of coding sequence. (C) Differences in kinetics of *AHA1* mRNA expression during heat-shock. Four independent experiments were performed. In each case, cultures of exponentially growing BY (red) and RM (black) cells were shifted from 30°C to 37°C, and cells were collected at indicated times after the transition. *AHA1* mRNA was quantified by quantitative real-time PCR with reverse-transcription relative to *ACT1* mRNA (a gene known to be stable after heat shock). Bars: +/− standard deviations.

Expression variability within a given strain background has also been studied in a broad sense by estimating the extent of variation across a large compendium of environmental conditions and/or specific genetic perturbations [36]. This “transcriptional plasticity” varies greatly among genes. For example, housekeeping genes display very low plasticity as they present stable expression across many conditions. This plasticity was previously associated with expression evolvability [36], [37] and nucleosome occupancy at promoter regions [39]. Using the values previously compiled [36], we found that SNEPs were enriched in genes displaying high transcriptional plasticity (Figure 4A). This enrichment was also visible when considering only SNEPs with strong effect (1.4-fold acetylation difference). Genes with at least one H3K14ac SNEP had significantly higher plasticity than genes with no SNEP (*t*-test *P* = 3.6×10^−7^ and 1.2×10^−6^ for BYac and RMac SNEP, respectively).

Finally, to see if SNEPs were more frequent among nucleosomes known to be evicted upon an environmental change, we used previous maps of nucleosome positioning in normal and stress conditions [26] and counted SNEPs among 147 remodeled nucleosomes and 61,623 unperturbed ones. Although this dataset represents only one environmental change and the remodeling of relatively few nucleosomes, a significant 1.7 fold enrichment of SNEPs was seen among these ‘mobile’ nucleosomes (*P* = 0.01, Chi-square).

### One SNEP is associated with differential gene activation upon stimulation

We reasoned that SNEPs could influence the dynamics of activation or repression. Intuitively, an acetylated nucleosome may be more rapidly evicted than a non-acetylated one upon promoter activation [40].

We noticed one SNEP where association with a differential dynamic response could be tested experimentally. A nucleosome contained a binding site for transcription factor Hsf1 (Heat Shock Factor 1) in the promoter region of the *AHA1* gene, which codes for a co-chaperone of Hsp90 known to be activated upon heat-shock [41]. This nucleosome had similar positioning in BY and RM but was acetylated at H3K14 in BY only (Figure 4B), while the DNA sequence of Hsf1-binding site was fully conserved between the two strains. Notably, other nucleosomes of the region were acetylated in both strains. We exposed BY and RM cells to heat shock and monitored *AHA1* mRNA by real-time quantitative RT-PCR (Figure 4C). Gene induction was unambiguously more pronounced in BY than RM. This marked difference was not observed when quantifying mRNA from three other *HSP* genes (*SSA3, FES1* and *CPR6*) lacking SNEP (Figure S8). This example illustrates how one SNEP can be associated with gene activation differences upon an environmental change.

## Discussion

We observed that nucleosome positioning at promoter regions was similar between two unrelated strains of *S. cerevisiae*. Because these strains have a large extent of transcriptional differences, this argues that differences in nucleosome occupancy profiles are not a major source of intra-species variation in gene expression.

In contrast, the epigenomic profile of H3K14ac was highly variable and this variability targeted specific nucleosomes. The presence/absence of a modification at a particular nucleosome in a given cell is, by definition, a discrete state. However, we observed quantitative acetylation differences that were often subtle (1.2 to 1.5 fold). This is likely due to high cell-to-cell heterogeneity and high dynamics of the acetylated state: all states from billions of cells were averaged in our samples, and no dynamical information was acquired over time. It is therefore important to interpret SNEPs as differences in the overall acetylation level across a cell population and not as a uniform epigenotype shared by all cells of the sample.

Natural epigenetic variation was previously reported at the level of methylated DNA (meDNA), particularly in plants [3], [14]. In this case also, differences were not necessarily discrete but often continuous. Important properties of SNEPs distinguish them from meDNA epi-polymorphisms. Methylated epi-alleles were predictive of lower gene expression [14] but SNEPs with reduced acetylation were not. In addition, no evidence was reported on a possible role of meDNA variation on the dynamics of gene activation.

Since histone-tail modifications are known to be highly reversible and dynamic, the basis and the origin of SNEPs remain to be further investigated. We observed that the two strains had different overall patterns of acetylation along genes, with a preferential acetylation near TSS and TES in the BY strain, while the RM strain had enriched acetylation in the second half of transcribed regions. This pattern difference accounted for many SNEPs and may result from trans-acting factors that act differentially in the two strains. However, 1806 SNEPs could not be attributed to this general inter-strain difference. Focusing on these SNEPs only, we looked again at their genomic distribution, their potential correlation to expression divergence and enrichment in genes with high plasticity (Figure S9). All conclusions made in our study were retrieved for this subset of SNEPs. Thus, the differential pattern of acetylation does not explain the general SNEP properties. Nucleosomal epi-polymorphisms may offer an alternative to irreversible nucleotide mutations. How BYac SNEPs accumulated at regulatory regions of conserved DNA is unclear. As mentioned above, it may occurred with the fixation of a *trans*-acting variation. Alternatively, accumulation may have occurred as a drift during laboratory culture conditions where fitness selection poorly applied. Future experiments examining a third wild strain will help determine if one of the two patterns is more ‘common’, if the stronger effect (acetylation fold-change) of BYac SNEP is peculiar, and if the abundance of SNEPs is similar in various pairwise comparisons of strains.

What is the origin of this epigenomic variability? E. Richards proposed a classification of epigenotypes based on their dependency on DNA variation [4], where the *obligatory*, *pure* and *facilitated* qualifications relate to genetic controls that are full, absent or incomplete, respectively. Following this terminology, *obligatory* SNEPs may result from genetic factors acting in *cis* or in *trans*. Known *cis*-regulations are exemplified by position effects of transposable elements, rDNA repeats or telomeric sequences. Trans-acting genotypes may reside in histone acetyl-transferase or de-acetylase machineries, or in upstream regulatory factors. Such *obligatory* SNEPs could have been fixed together with their genetic determinants. In contrast, if some SNEPs are *pure* (independent of genotype) they likely result from their direct selection. As SNEPs seem to relate to the dynamics rather than the steady-state levels of gene expression, this selection may act through the ability to respond to environmental changes (the Baldwin effect). Also, interactions between epigenotypes and genotypes are expected since histone acetylation can modulate the buffering of cryptic genetic variations [42], [43].

Acetylation of Lysine 14 of histone H3 at the beginning of protein-coding sequences has unambiguously been associated to high transcriptional activity in several studies [7], [27], [34]. It is therefore surprising that a preferential acetylation in one strain is not accompanied by a higher gene expression. This illustrates the complexity by which the various layers of inter-strain molecular differences are connected. Previous studies showed that DNA polymorphisms act on transcripts abundance in a complex manner [20], with a large extent of gene x environment effects [38] and that this genetic control was largely distinct from the control of protein levels [44]. Our results show that chromatin histone-borne modifications provide yet another layer of diversity, with non-trivial connections to genotypes and transcripts levels. SNEP identification and characterization provide a basis for population epigenetics of histone-borne modifications, and future quantitative epigenetics studies such as previously suggested [45], [46] will define the nature of these dependencies, and their relevance to the control of complex traits.

The abundance of SNEPs in highly-responsive genes and our observation that one SNEP correlated with the dynamics of gene activation upon stimulation suggest a contribution to gene x environment interactions. This is in full agreement with a previous report describing the contribution of H3K27me3 at the *FLC* locus of *Arabidopsis* to natural variation in cold-induced acceleration of flowering [17]. Except in such rare cases, gene-by-environment interactions have only been studied in the context of DNA variation. Integrating epigenotyping of histone marks in these investigations will likely better explain how individuals differ in their response to environmental changes.

In particular, attempts to predict and optimize the response to specific treatments is at the heart of personalized medicine. Chemical inhibitors of histone deacetylase are used in anti-cancer therapies and seem promising to fight other diseases [47], and ChIP-SEQ technologies [9] will soon provide clinicians with epigenotyping possibilities. Our results suggest that histone modification profiles of human individuals may greatly differ, with likely consequences on treatment outcome.

## Methods

### Nucleosomal DNA extraction and ChIP–CHIP

Yeast strains used were BY4716 (MATalpha, laboratory [48]) and RM11-1a (MATa, derived from wild isolate [20]). We processed six BY and six RM independent cultures for H3K14ac ChIP, plus three BY and three RM independent cultures for nucleosome mapping, totalling 18 microarray hybridizations. Cells were grown to exponential phase in synthetic medium with 2% glucose (SDall) as in Brem et al. [20]. We followed the protocol of Liu et al. [7] for both nucleosomal DNA isolation and ChIP, except that incubation time with micrococcal nuclease (Worthington Biochemical) prior to immunopurification was increased to 30 min at 37°C to obtain mononucleosomes. ChIP was performed using 3 µl of anti-H3K14Ac polyclonal antibody (Upstate, 07–353). For H3K14ac, efficiency was controlled by quantifying acetylation at the MAT locus by real-time quantitative PCR. This locus, as opposed to the silenced HML and HMR loci, is acetylated [49] and since BY and RM have opposite signs, we expect ChIP to be enriched for HMLalpha1 sequence in the case of BY and HMRa1 sequence in the case of RM (Figure S11). Real-time quantitative PCR was performed on a LightCycler 1.5 (Roche) using FastStart DNA Master Plus SYBR GREEN I kit (Roche). Primer pairs were 5′- AAATGTCTTGTCTTCTCTGCTC-3′ and 5′-ACTGTTG<@?show=[fo]?>CGCGAAGTAGT-3′ for HMLalpha1 and 5′-AAGAGCCCAAAGGGAAAATC-3′ and 5′-AGGCTTTGCTTTCTTCTA-3′ for HMRa1. ChIP and non-immunoprecipitated DNA fragments were linearly amplified using T-7 based in-vitro transcription as described previously [7] with few modifications. Briefly, the reaction mixture of 28.5 µl contained 18 µl template DNA, 5.2 µl 5x TdT buffer (Roche), 0.68 mM CoCl2 (Roche), 4.2 µM dTTP, 0.36 µM ddCTP, and 40 U terminal transferase (NEB). It was incubated at 37°C for 20 minutes and then stopped by adding 5 µl of 0.5 M EDTA (pH 8.0). Products were purified using Qiagen MinElute reaction cleanup kit and eluted in 20 µl nuclease free water, then concentrated to a 8 µl volume by Speed Vacuum centrifugation. The following were added: 0.6 µl of 25 µM T7- A_18_B primer, 1 µl of NEB buffer (2) and 0.4 µl of 5 mM dNTPs and the following thermal cycles were applied; 94°C for 2 min, decreasing to 35°C at -1°C/sec, hold down at 35°C for 2 min and decreasing to 25°C at -0.5°C/sec. Immediately after, 0.4 µl of Klenow enzyme (NEB) were added to the samples which were incubated at 37°C for 90 min. The reaction was halted by adding 5 µl of 0.5 M EDTA (pH 8.0). Products were purified using Qiagen MinElute reaction cleanup kit and eluted in 20 µl nuclease free water. The eluted samples were concentrated to a final volume of 5 µl. The IVT reaction mixture contained 5 µl nuclease free water, 2 µl 10X reaction buffer and 2 µl enzyme mix of MEGAshortscript® T7 kit (Ambion), 6 µl Labeling NTP mix from Affymetrix, and 5 µl T-7 tailed DNA and incubated at 37°C for 16 hrs. Amplified RNAs were purified using RNeasy Mini kit (Qiagen) and eluted in 50 µl of nuclease free water. RNAs (≥15 µg) were hybridized to GeneChip *S. cerevisiae* Tiling Array from Affymetrix [23] following manufacturer protocol.

### Heat shock

An isolated colony was picked to inoculate 4 ml SDall medium and incubated at 30°C with 220 rpm shaking for 12 to 16 h. This culture was used as a starter to inoculate 2 ml SDall medium at 0.1 OD_600_, which was grown for 6 hrs at 30°C with shaking. 1.5 ml of culture were then transferred to a microcentrifuge tube, incubated at 30°C in a water bath for 10 min, and incubated at 37°C for the times indicated on Figure 4. Cells were immediately harvested by centrifugation, re-suspended in 700 µl of TES buffer (10 mM Tris-HCl (pH 7.4), 10 mM EDTA and 0.5% SDS), snap frozen in liquid nitrogen and stored at −80°C. The experiment was conducted on BY and RM simultaneously, and repeated four times at different days. Total RNA was extracted using the following procedure: 700 µl of room temperature phenol was added to the cell extract, mixed well by vortexing and incubated at 65°C for 20 minutes. Extract was then snap frozen in liquid nitrogen for 1 min, thawed at room temperature, centrifuged at 13000 rpm for 5 min and the upper aqueous phase was transferred to a fresh microcentrifuge tube. Once again, 700 µl of room temperature phenol was added, mixed well and centrifuged at 13000 rpm for 5 min. The upper aqueous phase was transferred to a fresh tube, 700 µl of chloroform was added, mixed well and centrifuged at 13000 rpm for 5 min, the upper aqueous phase was purified using RNeasy mini kit (Qiagen) and eluted in 50 µl nuclease free water. RNA was precipitated by adding 50 µl of 3 M NaAc, and 1.25 ml of ice-cold ethyl alcohol to the purified samples followed by incubation at −20°C for 30 min. RNA was pelleted by centrifugation at 13000 rpm for 5 min, washed once with ice-cold 70% ethyl alcohol at 13000 rpm for 5 min and re-suspended in 50 µl of nuclease water. RNA concentration was quantified based on spectral absorbance using a NanoDrop ND-1000 Spectrophotometer. Reverse transcription and real time quantitative PCR were performed on a Stratagene MX3000P real-time PCR system using the Superscript III Platinum SYBR Green One-Step qRT-PCR kit from Invitrogen following manufacturer's protocol. Primers were 5′-GTCTGTTTCGTCCATTGAAGG-3′ and 5′- GTCCTTAGAGTCCACGTGTCC-3′ for AHA1, 5′-ATGGATTCTGAGGTTGCTGC- 3′ and 5′-TGGGAAGACAGCACGAGGAG-3′ for ACT1, 5′-GATGCAAAGAGATTAGAAACAGCG -3′ and 5′-GCCTTCCAACTCCTTTTGTCTA -3′ for SSA3, 5′-GATGAAGAACTACGTGCTGCTG-3′ and 5′-GCTTCGCAGACCATTGTCG-3′ for FES1 and 5′-CATTCCTTCTATCCATGGCC-3′ and 5′-GCTTCCCGTCCAAATGAG-3′ for CPR6. Amplification efficiencies and relative quantification of AHA1/ACT1, SSA3/ACT1, FES1/ACT1 and CPR6/ACT1 ratios were calculated as described by Pfaffl [50].

### Genome alignment

Genome sequences of S288c (isogenic to BY) and RM were downloaded in December 2007 from NCBI (ftp://ftp.ncbi.nih.gov/genomes/Saccharomyces_cerevisiae) and the Broad Institute (http://www.broad.mit.edu/annotation/genome/saccharomyces_cerevisiae/Home.html), respectively. The RM genome 8X assembly originates from whole genome shotgun and consists of 17 high-quality supercontigs (hqSC hence after) totalizing 11.7 Mb. The17 hqSC of RM were aligned on the 16 nuclear chromosome sequences of BY by using the *nucmer* algorithm implemented in MUMmer version 3.0 [51] with options *–maxgap = 1000 –mincluster = 50* considering BY chromosomes as the references and RM hqSCs as the queries. The output of *nucmer* was then filtered and formatted using the *delta-filter* and *show-coords* programs of the MUMmer package. At this stage, the output of our alignment pipeline consisted on a list of clusters of perfect matches between regions of BY chromosomes and RM hqSCs. We implemented an automatic rule to relate RM hqSCs to BY chromosomes by maximizing the coverage and alignment quality chromosome by chromosome. We then dynamically resolved overlapping clusters of perfect matches in order to get the longest aligned fragments of RM hqSCs along each BY chromosome. Visual inspections were also used in a few cases in order to define optimal boundaries of alignments. Detailed results of this genome alignment process as well as the hybrid shell/perl script used to do the genome alignment are available upon request.

### Sequence polymorphisms

Polymorphisms between BY and RM were detected by base substitution in the final alignment. Since base calling information were not available for RM sequences, we assumed that quality was reasonable and uniform along the RM genome sequence. From the 54,039 polymorphisms found, a few targeted repeated sequences (in both genomes as annotated by RepeatMasker (open-3.1.9, Smit, AFA, Hubley, R & Green, P. *RepeatMaskerOpen-3.0*. 1996–2004 http://www.repeatmasker.org) and were thus excluded, leading to a final core set of 52,280 polymorphisms consisting in 47,011 SNPs (90%), 2,448 insertions (4.5%) and 2,821 deletions (5.5%). These 2,448 insertions corresponded to 238,087 bp that were absent in RM while the 2,821 deletions corresponded to 80,349 bp absent in BY. This discrepancy between BY and RM insertions is mainly due to the heterogeneous content of Ty elements between both genomes (see below).

### Gene prediction and comparison

BY gene annotations were extracted from chromosomal features defined at NCBI website (ftp://genome-ftp.stanford.edu/pub/yeast/chromosomal_feature/saccharomyces_cerevisiae.gff). For RM, predicted gene set was downloaded from the Broad Institute website (http://www.broad.mit.edu/annotation/genome/saccharomyces_cerevisiae/Downloads.html). which were obtained using a combination of mapped ORFs from SGD predictions (http://www.yeastgenome.org), Glimmer [52] and GeneMark [53]. More details on this automated gene prediction pipeline are posted at http://www.broad.mit.edu/annotation/genome/saccharomyces_cerevisiae/GeneFinding.html#prediction. This original annotation file consisted of 5695 gene loci. 83 were dubious and discarded, as their annotated coding sequence did not code for a protein. 80 other predictions were also discarded because they fell in unaligned regions and could not be mapped to BY. Orthologs were identified using Blastp from WUBlast version 2.0 (Gish, W. (1996–2004) http://blast.wustl.edu) by aligning protein sequences of the 5532 remaining RM genes against the protein sequences of 6608 BY genes, and selecting reciprocal matches that fulfilled all following criteria: i) E-value <1.e-5, ii) percentage of identity >40% and iii) match length >75% of both protein sequences. This way, 5200 RM genes were called orthologs of BY genes, and 328 genes were RM-specific.

### Transcript boundaries

Transcript boundaries for BY genes were obtained from the complete published set of experimentally detected transcripts [23], [54] available at http://www.ebi.ac.uk/huber-srv/actinomycinD. Only transcript segments overlapping >50% of a non-dubious annotated coding region on the 5′ end were retained. This resulted in 4,714 verified transcription segments. We then mapped the BY transcript boundaries of ortholog genes on the RM genome, leading to 4,612 transcription segments on RM.

### Ty elements

We extracted the DNA sequences of the 50 active Ty elements annotated in the BY genome (as defined in the NCBI chromosomal features, leading to 31 Ty1, 13 Ty2, 2 Ty3, 3 Ty4 and 1 Ty5) and blasted them onto the 17 RM supercontigs. We found 10 matches on RM with 10 Ty2 elements (percentage of identity >90% and match length >95%). No match was found with any other Ty elements even after relaxing these criteria, indicating that Ty elements populating the RM genome are Ty2 only. Of these 10 Ty2 elements found in RM, 3 were located at the same place in BY, 6 were located elsewhere in RM (leading to 6 deletions (insertions) in BY (RM)) and 1 replaced a Ty1 element of BY.

### Other annotations and data used

*Conserved Regulatory Sites* (CRS) [35] were downloaded from (http://fraenkel.mit.edu/improved_map/p001_c3.gff). *Conserved Non-Coding Sites* were obtained from the UCSC website (http://genome.ucsc.edu/), using data from table phastConsElements for track MostConserved, and were then defined as the intersection between conserved regions and non-coding regions. Gene expression values of Brem et al. [20] were downloaded from NCBI GEO site (http://www.ncbi.nlm.nih.gov/sites/entrez?db=gds, dataset GSE1990). Regions where nucleosome(s) were remodeled upon heat-shock were extracted from whole-genome nucleosome maps of Shivaswamy et al. [26] filtered for nucleosomes of normalized score <0.2. They were defined as chunks of at least 145 consecutive nucleotides (average size of a nucleosome) covered by a nucleosome in only one condition (unstressed or heat-shock). Using our BY atlas of nucleosome positions (see below), 147 nucleosomes (∼0.2%) were then said to be remodeled if they lied entirely within a remodeled region.

### Microarray analysis

The 25 bp array probes were mapped on BY and RM genomes using MUMmer. For each strain, we kept only probes that had unique perfect match on the genome (2,801,885 probes for BY and 2,570,638 probes for RM, overlap: 2,491,913 probes). For nucleosome mapping (see below) we used only the 3 array replicates per strain. Since informative probe sets differed between the two strains, normalization was done separately for each strain and a log2 transformation of probe signals was applied before normalization [55]. For SNEP identification (see below), only the subset of RM probes that can be mapped within ±3 bp of the corresponding probe in BY was kept to insure that at every positions the same probe is used between the two strains. In addition, since any DNA polymorphism would bias hybridization efficiency, we discarded probes containing at least one BY/RM polymorphism, keeping a final core set of 2,356,676 probes. Then the full dataset (18 arrays) was log2-transformed and quantile-normalized all together using only this set of probes with dual perfect match.

### Nucleosome mapping

We positioned nucleosomes in each strain separately using only the 3 dedicated replicates per strain. We then implemented a custom version of the Hidden Markov Model devised by Yuan et al. [25]. Our HMM implementation was similar to the one used by Lee et al. [24], except than we did not train the HMM on specific regions but used sliding windows as in Yuan et al. in order to remove unpredictable trends in the hybridization signal. Thus, independent run of the HMM were successively applied in window of 1 kb (i.e. ∼250 probes) all along the genome. The model parameters and posteriors of all windows containing a fixed probe were then averaged and used for a global computation of both state probabilities and most-likely states (among well-positioned nucleosome, fuzzy nucleosome and linker). As we used only probes with unique and perfect matches and that regions with high SNP density between BY and RM can lead locally to low probe coverage in RM, we also allowed the HMM to deal with missing data. State probabilities and most-likely states of “missing probes” were computed in the same way than for observed probes, taking advantage of neighboring observed information.

### Nucleosome alignment

The most-likely nucleosome occupancy profiles of the two strains (as obtained from the Viterbi algorithm on each chromosome) were aligned according to the genome alignments. The BY genome was the reference and the positions of RM nucleosomes on this reference were obtained from the coordinates of RM nucleosomal sequence fragments on the BY genome. Once RM nucleosomes were “mapped” on BY, we used a dynamic algorithm to align RM and BY nucleosomes. The algorithm works chromosome-by-chromosome as follows:

1. Assumption: two nucleosomes are said to be “unambiguously aligned” if the distance between their midpoints is lower than half of their average size.
2. Initiation: find all unambiguously aligned nucleosomes along the chromosome.
3. Recursion: between two consecutive unambiguously aligned nucleosomes:
  - if there is no unaligned nucleosome in the interval, then go to the next unambiguously aligned nucleosome.
  - else:
    - Align nucleosomes by minimizing physical distance between aligned pairs.
    - The remaining nucleosome(s) are considered as insertion(s) (if they are BY nucleosomes) or deletion(s) (if they are RM nucleosomes).
    - Go to the next unambiguously aligned nucleosome.

More stringent assumptions can be used to define “unambiguously aligned” nucleosomes without significant changes on the final results (data not shown). This strategy aligned 64,294 nucleosomes between the two strains, i.e. ∼95% of RM nucleosomes. Finally, in order to evaluate locally the quality of our alignment, for each pair of aligned nucleosomes we computed their likelihood as L(aligned)  =  B_n_.R_n_ where B_n_ and R_n_ are the probabilities that the corresponding probes belong to a nucleosome in BY and RM, respectively. Similarly, we computed the likelihood for insertion and deletion of a nucleosome (with respect to BY) as L(insertion)  =  B_n_*R_l_ and L(deletion)  =  B_l_*R_n_ where B_l_ and R_l_ are the probabilities that the corresponding probes belong to a linker in BY and RM, respectively. Within each strain, the probability of each state (nucleosome or linker) was derived from probe-level posterior probabilities (as estimated by our HMM, and merging fuzzy and well-positioned nucleosome states into a single class) averaged over probes covering the target region.

### Promoter clustering

To generate Figure 1B and 1C, we first divided the +/−300 bp region around the TSS of each transcript into 60 bins of equal size (10 bp). We then computed the average nucleosome occupancy in each bin by averaging the posterior probabilities to be a nucleosome (output by our HMM and summing posteriors from well-positioned and fuzzy nucleosomal states) of the probes within the bin. We then applied K-means clustering (with *kmeans* function implemented in the base package of R) using the Euclidean distance metric and 25 repetitions for each number of cluster tested (1< = K< = 10). Visual inspection together with standard clustering validity measures (e.g. ratio of variance within clusters and variance between clusters) were used to choose the optimal number of clusters (K = 6).

### SNEP identification

To screen for SNEPs, we considered only pairs of aligned nucleosomes sharing at least 15 microarray probes, which was the case of 97% of aligned pairs. Following previous linear models validated for transcripts quantification [56], we applied to each pair the following analysis of variance (ANOVA): 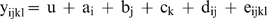
where y_ijkl_ is the log2 normalized hybridization intensity of probe *k* in replicate *l* for strain *i* (BY or RM) in experiment type *j* (nucleosome positioning or ChIP-ChIP), u is the global mean of the signal, a_i_ is the strain effect (BY or RM), b_j_ is the experiment type effect (nucleosome positioning or ChIP-Chip), c_k_ is the probe effect, d_ij_ is the interaction term between strain and experiment type and e_ijkl_ is the residual. We reasoned that if a nucleosome carries the modification then the corresponding DNA is present in both ChIP and nucleosomal positioning samples and signal expectancies should not differ between experiment types (b_j_ = 0). However, if a nucleosome carries the modification in only one strain, then a significant interaction should be seen between experiment type and strain (d_ij_ ≠ 0) (Figure S12). We therefore used an *F*-statistic to test (H_0_: d_ij_ = 0 vs. H_A_:d_ij_ ≠0) and derived nucleosome-level *P*-values. A striking enrichment of low *P*-values was observed (Figure S13). We applied the false discovery rate (FDR) control procedure [28] to compute a genome-wide cutoff from our sorted vector of 58,694 *P*-values.

### Data accession numbers

EMBL ArrayExpress accession number E-MEXP-1777. Processed data files and the C source code of *NucleoMiner* (for Unix-based platforms) are available on our web site http://www.ens-lyon.fr/LBMC/gisv/snep/

## Supporting Information

Figure S1 Nucleosome alignment conservation. For 63,706 pairs of BY/RM aligned nucleosomes, we computed the distance between the two midpoints (black histogram and axis), as well as the fraction of the BY nucleosome that overlapped the RM nucleosome (red histogram and axis).
(0.22 MB AI)

Figure S2 The fraction of nucleosomal DNA was computed in 2.5-Kb sliding windows across the genome, excluding windows containing insertions or deletions of 5 bp or more. (A) Histogram of the distribution of BY - RM occupancy differences among all windows. Most differences are small, and few extremes are detected. (B) Genomic location of windows with extreme values (first and last 1/1000 quantile, red  =  greater than 0.82, blue  =  lower than -0.85).
(0.35 MB AI)

Figure S3 Global nucleosomal occupancy at TSS and TES. Neighborhood regions of TSS (left panels) and TES (right panels) were divided in 10 bp bins. Probes were assigned to bins according to the position of their midpoint. For all probes, the HMM posterior probabilities to be in a nucleosomal state (delocalized or well-positioned) were summed. For every bin, this cumulated probability was averaged across all probes of the bin. Profiles shown are average profiles across all genes for BY (upper panels) and RM (lower panels). Note that coordinates of transcripts were determined in the BY strain background [23] and may slightly differ in RM. This is likely to explain the fact that depletions at TSS and TES, as well as oscillations downstream TSS appear less pronounced in RM than in BY.
(0.29 MB AI)

Figure S4 The degree of acetylation differences in SNEPs is shown by their cumulative distribution of ‘foldchange’ values, which correspond to *(acBY/nucBY)/(acRM/nucRM)* for BYac SNEPs and its inverse for RMac SNEPs. Most SNEPs show folchanges between 1.2 and 1.5, and BYac SNEPs are associated with higher acetylation differences than RMac SNEPs.
(0.20 MB AI)

Figure S5 Scheme of nucleosome organization in the region of the NDE2 gene. Rectangles represent nucleosomes, colored according to the mean *log(ac/nuc)* value across all probes of the nucleosome. SNEP detection (-log10(P-value)) is indicated above each nucleosome, with significance cutoff indicated as a dashed line. Brown boxes, coding sequences.
(0.27 MB AI)

Figure S6 For all panels, BYac and RMac SNEP frequencies were counted among nucleosomes located in each gene (coding region plus and minus 250 bp), and averaged in 500-genes sliding windows (y-axis). Genes were sorted (x-axis) either by their BY/RM foldchange in expression, which showed SNEP enrichments at both extremities (A), or by their Expression Divergence (Tirosh et al. [36]) (B), or by their mutational variance (*Vm* values of Landry et al. [37]) (C), which showed SNEP enrichment at high values.
(0.42 MB AI)

Figure S7 For every gene, we considered the *P*-value (significance) of the genotype x interaction term of the ANOVA model of Smith & Kruglyak 2008 [38]. We then ranked all genes according to sg * P, where *sg* is the sign of the interaction term. These ranks are reported on the x-axis. SNEP frequency (y-axis) was computed in sliding windows as in Figure S6.
(0.29 MB AI)

Figure S8 Kinetics of mRNA expression during heat-shock for three genes lacking SNEPs. Three genes (*SSA3, FES1*, and *CPR6*) were selected based on the following criteria: known to be induced upon heat-shock (from the data of Gasch et al. 2000 Mol. Biol. Cell. 11: 4241–4257), possess an HSF1 binding site in the promoter with no BY/RM polymorphism in it, do not have H3K14ac SNEP, do not have a marked BY/RM difference in expression in non-induced cultures. RNA levels of these three genes were measured by real-time quantitative RT-PCR on the same samples as for *AHA1* on Figure 4. Bars: ± standard deviations.
(0.25 MB AI)

Figure S9 Properties of SNEPs disagreeing with the patterns of Figure 2D. To distinguish SNEPs that support the general patterns of Figure 2D (BYac SNEP around TSS and TES and RMac SNEPs within transcribed region), three regions were defined. R1: from −500 bp to TSS, R2: from TSS to TES and R3: from TES to +400 bp. We then flagged all BYac SNEP falling in R1 and R3, and all RMac SNEPs falling in R2. For nucleosomes that did not fall entirely in the region, we flagged them if >75% of their length overlapped the region. 1,806 SNEPs remained unflagged and were used to re-draw the main figures of the article. Please see the main figures of the article (that reflect all SNEPs) for legends.
(1.91 MB AI)

Figure S10 Absence of correlation between H3K14 acetylation differences and amplitudes of gene expression differences. The display is similar as in Figure 3A, except that expression fold changes are presented in the x-axis instead of statistical significance of expression differences.
(1.47 MB AI)

Figure S11 Internal controls provided by the MAT locus. (A) Scheme of the *MAT*, *HML* and *HMR* loci. Nucleosomes are acetylated at the MAT locus but not at the two silenced *HML* and *HMR* loci [49]. Strains of type alpha, such as BY, have a single copy of the *a1* sequence located at *HMR* and two copies of the *alpha1* sequence: one at *HML* and the other at *MAT*. Strains of type a (such as RM) differ only at the *MAT* locus containing *a1* instead of *alpha1*. Thus, H3K14 acetylated chromatin fractions are expected to be enriched in alpha1 DNA for BY and a1 DNA for RM. (B) Dosage of alpha1 and a1 sequences in ChIP fractions by real-time quantitative PCR (absolute quantification) as explained in methods. (C) Abundance of *alpha1* and *a1* sequences in the two strains was compared from raw intensity values of 23 and 22 microarray probes, respectively, interrogating the same fragments as those amplified in (B). Ordinate values correspond to log(*acBY/nucBY*) - log(*acRM/nucRM*) for each probe, where *acX* and *nucX* represent mean intensities across replicates of ChIP and mapping experiments, respectively, performed on strain X.
(0.29 MB AI)

Figure S12 SNEP detection. The data from four nucleosomes is presented. *nuc*, nucleosome mapping experiments. *H3K14ac*, ChIP experiments. Normalized hybridization intensities y_ijkl_ (see Methods) were corrected for probe effect by subtracting the mean signal probe value c_k_. Each box summarizes about 100 and 200 data points for *nuc* and *H3K14ac* categories, respectively. Acetylated nucleosomes are expected to produce *H3K14ac* and *nuc* signals of similar intensities.
(0.26 MB AI)

Figure S13 Histogram of SNEP *P*-values across all nucleosomes interrogated by the ANOVA model described in the Methods.
(0.25 MB AI)

Text S1 Densities of BY/RM SNPs in nucleosomal versus linker DNA.
(0.05 MB PDF)
