# Supplementary material for: Natural Single-Nucleosome Epi-Polymorphisms in Yeast
Source: PLoS Genet. 2010 Apr 22;6(4):e1000913. doi: 10.1371/journal.pgen.1000913 (PMC2858693; doi:10.1371/journal.pgen.1000913)
Supplement: Text S1 — Densities of BY/RM SNPs in nucleosomal versus linker DNA. (0.05 MB PDF) [file pgen.1000913.s014.pdf]

SUPPLEMENTARY TEXT

Densities of BY/RM SNPs in nucleosomal versus linker DNA

Using our nucleosome positioning dataset, we observed a higher frequency of BY/RM SNPs in linker DNA than in nucleosomal DNA when counting SNPs across the entire genome (Table 1). This observation contradicts two previous reports, where higher evolutionary rates were observed in nucleosomal DNA [2,3]. To compare the output obtained from another nucleosome positioning dataset, we re-counted these fractions using the dataset of Lee et al. [1], and obtained very similar results (Table 1).

Warnecke et al. PLoS Gen 2008 also used the atlas of Lee et al. but they focused on protein coding sequences. When using the same list of 5788 coding sequences, we found more BY/RM SNPs in nucleosomes than in linkers, and results were consistent across the two atlases. So the apparent contradiction reflected strong differences between coding and non-coding DNA (Table 1).

Table 1: Fraction of BY/RM SNPs in nucleosomal and linker DNA, measured directly from fitted HMM model.

Method: Direct Counting				
	All Genome		Coding sequences considered in Warnecke et al.	
	Using Atlas of Lee et al.	Using Atlas of this study	Using Atlas of Lee et al.	Using Atlas of this study
Linker	0.0045	0.0044	0.00324	0.00365
Well-positioned	0.0041	0.0040	0.00388	0.00373
Fuzzy and Well-positioned	0.0039	0.0040	0.00371	0.00368

Washielt et al.[3] used the dataset of Whitehouse et al. [4] and a different method to estimate mutation rates in nucleosomal versus linker DNA. Note that this dataset differs from Lee's atlas and ours by both methodology (different microarrays) and positioning inference (different model). The method used by Washielt et al. to distinguish nucleosomal from linker DNA was based on these definitions:

Nucleosomal = 147 nucleotides centered on the 'peak' position of the nucleosome

Linker = 10 nucleotides upstream and downstream each nucleosomal region.

We applied this method to count the fraction of BY/RM SNPs in both categories, across the entire genome (Table 2). When using our atlas or the one of Lee et al., we centered the 147nt window on the middle of each inferred nucleosome, regardless of the predicted size of the nucleosome. A slight enrichment of SNPs was seen in nucleosomes. This enrichment was more pronounced when using the Whitehouse dataset.

So the apparent contradiction reflected strong differences in the methodology used to distinguish the two categories of DNA.

Table 2: Fraction of BY/RM SNPs in nucleosomal and linker DNA, measured by centering a fixed-size window on mid-positions of nucleosomes.

Method: Washielt et al.			
	Using Atlas of Lee et al.	Using Atlas of this study	Using Atlas of Whitehouse et al.
Linker	0.0038	0.0039	0.0037
Nucleosome	0.0039	0.0040	0.0040

REFERENCES:

1. Lee W, Tillo D, Bray N, Morse RH, Davis RW, et al. (2007) A high-resolution atlas of nucleosome occupancy in yeast. Nat Genet.
2. Warnecke T, Batada NN, Hurst LD (2008) The impact of the nucleosome code on protein-coding sequence evolution in yeast. PLoS Genet 4: e1000250.
3. Washietl S, Machne R, Goldman N (2008) Evolutionary footprints of nucleosome positions in yeast. Trends Genet 24: 583-587.
4. Whitehouse I, Rando OJ, Delrow J, Tsukiyama T (2007) Chromatin remodelling at promoters suppresses antisense transcription. Nature 450: 1031-1035.
